# Ophthalmovigilance in pharmacotherapy of presbyopia

**DOI:** 10.5935/0004-2749.2023-0196

**Published:** 2023

**Authors:** Marianne Levon Shahsuvaryan

**Affiliations:** 1 Department of Ophthalmology, Yerevan State Medical University, Yerevan, Armenia

Dear Editor,

Presbyopia as an age-related vision condition, characterized by insufficient accommodative amplitude for clear near vision, is the most common ocular affliction worldwide. It has been estimated that the number of persons with presbyopia will reach 1.9 billion by 2050.

The millennial-minded approach in presbyopia requires independence from corrective lenses considering the quality of life. To achieve this goal, presbyopia is currently considered a druggable target, specifically in a topical route by eyedrops. Multiple approaches have been developed; however, pharmacotherapy for presbyopia starts from repurposing pilocarpine, which has been used since 1875 for glaucoma therapy.

In October 2021, the Food and Drug Administration (FDA) approved pilocarpine hydrochloride 1.25% eye drops (VUITY) as a first-line treatment for presbyopia based on pupil constriction and contraction of the ciliary body^(1)^.

Two phase III randomized, placebo-controlled trials showed that statistically significantly more persons treated with self-administered, once-daily pilocarpine achieved 3 lines or more on a reading chart without losing more than one line in distance visual acuity at day 30. No serious adverse events were reported. The most common adverse events were transient headache and red eye, occurring in more than 5% of the participants.

Recently, the FDA has approved a twice-daily dosing option of VUITY intended to extend the duration of effect to 9 h, based on results from the double-masked Phase 3 VIRGO trial^(2)^. The frequency of headache and eye irritation was similar to that during the once-daily use; however, note that ocular adverse reactions, such as visual impairment, eye pain, blurred vision, and vitreous floaters, were reported in 1%-5% of the participants.

Real-world experience underscores the need for comprehensive evaluation of the adverse effects, which may not become evident at the 14- and 30-day endpoints.

The undesirable side effects of pilocarpine include chronic inflammation, fixed pupil, posterior synechiae, inadequate dilation for detailed retinal exam and cataract surgery, pigment dispersion, myopic shift, and lens opacifications that deteriorate vision in dim illumination. In such cases, driving at night will be dangerous, particularly when taken twice-daily. Pilocarpine can cause an acute angle closure attack, particularly in persons with a narrow anterior chamber angle.

Since the recent FDA approval of VUITY and its commercial availability, some papers and case reports have reported cases of retinal detachment^(3,4)^ and vitreofoveal traction^(5)^. A recent multicenter case series identified three cases of retinal detachment in two patients using topical pilocarpine as treatment for presbyopia^(3)^. The researchers hypothesized that the drug may cause anterior lens migration, which could transmit tractional forces on the retina.

Another two cases of retinal detachment occurring within 10 days of starting pilocarpine 1.25% drops were reported by Eton et al.^(4)^.

Amarikwa et al.^(5)^ presented a case of a 65-year-old female patient who developed vitreomacular traction immediately following the first administration of VUITY.

With recent case reports showing rare but serious adverse effects with the use of VUITY (pilocarpine hydrochloride ophthalmic solution, Allergan), the company expanded its warning label^(6)^.

## Retinal detachment

Rare cases of retinal detachment and retinal tear have been reported with miotics, including VUITY.

Individuals with preexisting retinal disease are at an increased risk. Therefore, retinal examination is advised in all patients before the initiation of therapy.

Patients should be advised to seek immediate medical care with a sudden onset of flashing lights, floaters, and/or vision loss.

Special attention should be paid to patients who be came emmetropic after photorefractive surgeries correcting myopia, particularly postlaser-assisted *in situ* ke-ratomileusis patients with permanent retinal alterations.

To overcome this challenge, a promising approach in pharmacotherapy for presbyopia using pilocarpine is directed toward the use of a lower concentration of miotics.

Recently, the phase 3 Near-1 and Near-2 clinical trials compared a preservative-free drop consisting of 0.4% pilocarpine delivered in a proprietary vehicle CSF-1 (Orasis Pharmaceuticals) with vehicle and reported visual benefits with clinically meaningful near vision improvement in CSF-1 recipients^(7)^. However, note that to evaluate the long-term safety of this treatment approach, Orasis plans to conduct such a study.

In summary, ophthalmologists must be vigilant and should carefully perform presbyopia screening to determine persons eligible for the prescription of pilocarpine. Furthermore, they must follow the Hippocratic Oath, “First Do No Harm”.

## References

[1] Abbvie. News Center (2021). VUIT TM (Pilocarpine HCI ophthalmic solution) 1.25%, the First and Only
FDA-approved eye drop to treat age-related blurry near vision (Presbyopia),
is now available. [Internet].

[2] Kannarr S, El-Harazi SM, Moshirfar M, Lievens C, Kim JL, Peace JH (2023). Safety and efficacy of twice-daily pilocarpine HCI in presbyopia:
The Virgo Phase 3, Randomized, Double-Masked, Controlled
Study. Am J Ophthalmol.

[3] Al-Khersan H, Flynn HW Jr, Townsend JH. (2022). Retinal detachments associated with topical pilocarpine use for
presbyopia. Am J Ophthalmol.

[4] Eton EA, Zhao PY, Johnson MW, Rao RC, Huvard MJ. (2022). Rhegma-togenous retinal detachment following initiation of
pilocarpine hydrochloride ophthalmic solution 1.25% for treatment of
presbyopia. Retin Cases Brief Rep.

[5] Amarikwa L, Michalak SM, Caul S, Mruthyunjaya P, Rahimy E. (2022). Vitreofoveal traction associated with pilocarpine for presbyopia.
Ophthalmic Surg Lasers Imaging. Retina.

[6] Drug Label Information. VUITY- pilocarpine hydrochloride solution/drops.
5.2. Risk of retinal detachment [Internet].

[7] Orasis Pharmaceuticals (2022). Orasis Pharmaceuticals Announces Positive Phase 3 Topline Results of
novel eye drop candidate, CSF-1, for the treatment of presbyopia.

